# Taeniid cestodes in a wolf pack living in a highly anthropic hilly agro-ecosystem

**DOI:** 10.1051/parasite/2021008

**Published:** 2021-02-05

**Authors:** Fabio Macchioni, Francesca Coppola, Federica Furzi, Simona Gabrielli, Samuele Baldanti, Chiara Benedetta Boni, Antonio Felicioli

**Affiliations:** 1 Department of Veterinary Sciences, University of Pisa Viale delle Piagge 2 56124 Pisa Italy; 2 Department of Public Health and Infectious Diseases, Sapienza University of Rome Piazzale Aldo Moro 5 00185 Rome Italy

**Keywords:** Anthropic areas, *Canis lupus*, *Echinococcus granulosus* s.s., *Taenia hydatigena*, Helminths, Parasites

## Abstract

The Italian wolf population in human-modified landscapes has increased greatly in the last few decades. Anthropisation increases the risk of transmission of many zoonotic infections and in this context, control of taeniid cestode species needs to be addressed from a One Health perspective. Predator-prey interactions are at the root of taeniid cestode transmission, and the wolf plays a key role in the maintenance and transmission of taeniids. To date, all available data on the taeniids of wolves in Italy refer to populations living in a wild habitat. Between 2018 and 2019, we investigated taeniids in a wolf pack living in a highly anthropic hilly agro-ecosystem. Thirty-eight faecal samples were collected and analysed, 4 of which were also genetically characterised for individual wolves and belonged to three different animals. Samples collected were analysed microscopically and by molecular analysis in order to identify the taeniid species. Taeniid eggs were detected in 34.2% (13/38) of samples. Within samples positive to taeniid eggs only *Echinococcus granulosus* s.s. and *Taenia hydatigena* were identified in 26.3% and 10.5% of the samples, respectively. On microscopic examination, *Capillaria* spp., Ancylostomatidae and *Toxocara canis* eggs, *Crenosoma vulpis* larvae, and coccidian oocysts were also found. The combination of low biodiversity of taeniid species with a high occurrence of *E. granulosus* s.s. recorded in this study could be the consequence of a deeper link occurring between wolves and livestock in human-modified landscapes than in wild settings.

## Abstract (fr)

La population de loups italiens dans les paysages modifiés par l’homme a considérablement augmenté au cours des dernières décennies. L’anthropisation augmente le risque de transmission de nombreuses infections zoonotiques et, dans ce contexte, le contrôle des espèces de cestodes Taeniidae doit être abordé dans une perspective One Health. Les interactions prédateurs-proies sont à la base de la transmission des Cestodes Taeniidae, et le loup joue un rôle clé dans le maintien et la transmission des Taeniidae. À ce jour, toutes les données disponibles sur les Taeniidae du loup en Italie se réfèrent aux populations vivant dans un habitat sauvage. Entre 2018 et 2019, les Taeniidae d’une meute de loups vivant dans un agro-écosystème vallonné hautement anthropique ont été étudiés. Trente-huit échantillons fécaux ont été collectés et analysés, dont quatre ont également été génétiquement caractérisés pour des loups individuels et appartenaient à trois animaux différents. Les échantillons prélevés ont été analysés au microscope et par analyse moléculaire afin d’identifier les espèces de Taeniidae. Des œufs de Taeniidae ont été détectés dans 34,2 % (13/38) des échantillons. Dans les échantillons positifs aux œufs de Taeniidae, seul *Echinococcus granulosus s.s.* et *Taenia hydatigena* ont été identifiés dans 26,3 % et 10,5 % des échantillons, respectivement. Lors de l’examen microscopique, des œufs de *Capillaria* spp., d’Ancylostomatidae et de *Toxocara canis*, des larves de *Crenosoma vulpis* et des oocystes de coccidie ont également été trouvés. La combinaison d’une faible biodiversité d’espèces de Taeniidae avec une forte occurrence d’*E. granulosus s.s.* rapportées dans cette étude pourrait être la conséquence d’un lien plus profond entre le loup et le bétail dans les paysages humains que dans les paysages sauvages.

## Introduction

The family Taeniidae includes four genera: *Taenia* Linnaeus, 1758, *Echinococcus* Rudolphi, 1801, *Hydatigera* Lamarck, 1816), and *Versteria* Nakao, Lavikainen, Iwaki, Haukisalmi, Konyaev, Oku, Okamoto & Ito, 2013, which parasitise both mammals and humans [33, 47, 59]. Within the genera *Taenia* and *Echinococcus*, *Taenia solium*, *Taenia saginata*, *Echinococcus granulosus s.l*. and *Echinococcus multilocularis* are important pathogens causing food-borne zoonotic infections worldwide [15]. Tapeworm transmission is based on indirect domestic, semi-domestic and wildlife cycles involving various mammalian hosts including: (i) wild or domestic herbivores (prey), or (ii) wild or domestic canids or felids (predators) [33]. Occasionally, certain other zoonotic tapeworm species can also infect humans [33].

Predator–prey interactions are at the root of taeniid transmission, in which predators are the definitive hosts, while the prey is the intermediate host. This is referred to as a multi-host trophically-transmitted parasite system [3].

Among definitive hosts, the wolf (*Canis lupus*) plays a key role in the maintenance and transmission of several Taeniidae and could serve as a model species to better understand prey–predator and host–parasite dynamics [20]. Due to its top position in the wild trophic chain, the wolf hosts a wide gastrointestinal parasite community, which changes in relation to its diet [20].

As an opportunistic predator, the wolf selects its preys according to their local abundance, accessibility and vulnerability [39–41]. In Italy, the wolf’s diet is mainly based on wild ungulates such as wild boar (*Sus scrofa*), roe deer (*Capreolus capreolus*), red deer (*Cervus elaphus*) and fallow deer (*Dama dama*), but it also preys upon livestock, especially goats (*Capra hircus*), sheep (*Ovis aries*) and calves (*Bos taurus*) [16, 32, 39, 42].

The Italian wolf population (*C. lupus italicus*, Altobello 1921), was in strong decline until the 1970s [60]; however, in the last few decades it has undergone natural re-expansion throughout the Alps and Apennines and in human-modified environments [14, 21].

The wolf’s legal protection in Italy, which was established in 1976, together with changes in the ecology of mountain areas (e.g. decrease in human density and an increase in wild ungulates) as well as the natural reforestation of these areas, have promoted the wolf’s re-colonisation of its historical distribution range [6, 8]. At the same time, the establishment of human activities in natural habitats has led to an increase in wolf–human conflicts due to predation on livestock [41] and wolf/domestic animal/human contact, with a high risk of pathogen transmission [29, 56]. Increased anthropisation may be a risk for the transmission of many zoonotic infections; furthermore some taeniid species are good examples to discuss the One Health perspective [29].

The Taeniidae of the Italian wolf population are still scarcely known and all the available data have been obtained from investigations performed in natural and semi-natural landscapes in the Apennines and Southern Alps [11, 18, 22–24, 37, 44, 49]. In these areas, *T. hydatigena* was the most frequently detected taeniid species in wolves, while *T. multiceps*, *T. krabbei*, *T. ovis*, *E. multilocularis* and *E. granulosus s.s*. were also found in low frequency [22, 23, 37, 49].

Within the *E. granulosus s.l.* species cluster, *E. granulosus s.s.* and *E. canadensis* are mainly detected in wolves [23, 49, 57]. *Echinococcus granulosus s.s.*, the major cause of cystic echinococcosis (CE) in humans in the world [1], is highly endemic in the Mediterranean basin, particularly in rural livestock-raising areas [10]. In these areas, the prevalence of *E. granulosus s.l.* in wolves is closely linked to the semi-domestic cycle as a consequence of the high prevalence of cystic echinococcosis in sheep [24, 49].

Since no data are available on the Taeniidae population in wolves living in newly colonised, highly anthropic areas, the aim of this study was to investigate the Taeniidae in a wolf pack living in a highly anthropic hilly agroecosystem using non-invasive sampling. We predicted that in such areas, Taeniidae species from domestic intermediate hosts would be the most frequently recorded species associated with low taeniid biodiversity as a consequence of a deeper link occurring between wolves and livestock in human-modified landscapes than in wild environments.

## Materials and methods

### Study area

The study was carried out in a sub-urban, hilly area in Crespina-Lorenzana and Lari-Casciana Terme (43.551370°–10.551708°) in the Province of Pisa (central Italy). The study area of 900 ha is a highly anthropic, sub-urban, hilly, fragmented, woody agro-ecosystem where a wide variety of wildlife mammals live, such as crested porcupines (*Hystrix cristata*), wild boar (*Sus scrofa*), roe deer (*Capreolus capreolus*), pine martens (*Martes martes*), stone martens (*Martes foina*), European polecats (*Mustela putorius*), badgers (*Meles meles*), hares (*Lepus europaeu*s), eastern cottontails (*Sylvilagus floridanus*), wild rabbits (*Oryctolagus cuniculus*), red foxes (*Vulpes vulpes*), and a large variety of small mammals. In the study area, small, woody areas are interspersed with human settlements, which lead to intensive interactions between the wildlife and productive and recreational activities of humans (i.e. sheep farming, forest cutting, trekking, cycling, horse riding, bird watching, and hunting). The study area includes 18 villages, with an average human density of 134.08 people/km^2^. Extensive or semi-extensive sheep and cattle farming are the main husbandry economic activities in this area, mainly consisting of small farms (120–200 animals).

### Sample collection and parasitological analysis

**Figure 1:**
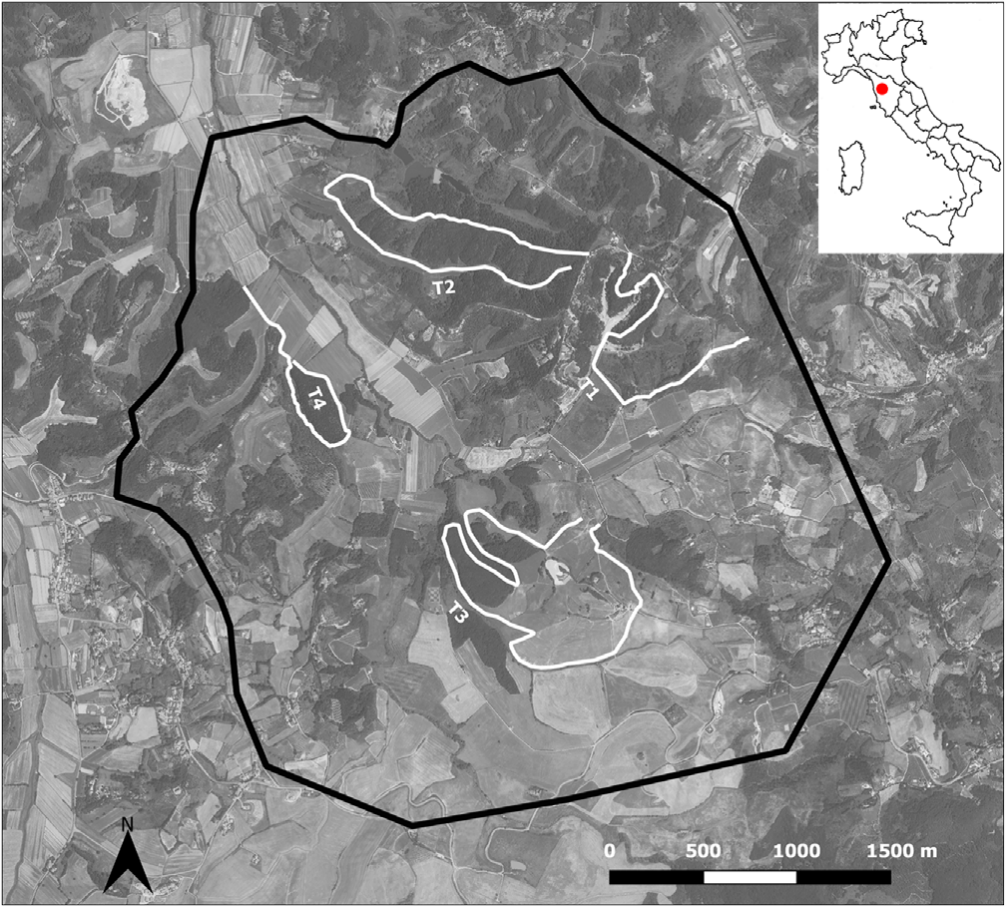
The sub-urban, hilly study area in Crespina-Lorenzana and Lari-Casciana Terme (black line border). The white lines indicate the four transects (T1–T4) where wolf faecal samples were collected. The location and country map are shown in the inset. The study area image was created using QGis 2.18 software.

The parasitological investigations were carried out on wolf faecal samples collected between October 2018 and December 2019 within a larger non-invasive genetic monitoring project on a wolf pack (6–10 individuals), living in the study area. Whenever possible, faecal samples collected for genetic individual identification were also used for parasitological analysis. The faecal samples were collected along four transects (3 SD 1.2 km), for a total length of 12 km. Transects were randomly chosen within existing footpaths in the zones continuously monitored by camera-traps where transits and signs of presence (i.e. faeces, tracks, footprints, predations) of wolves were regularly recorded (Fig. 1).

Each transect was covered twice a week and all faeces were sampled, geolocated using GPS trackers and mapped in QGis 2.18 software. Since stray dogs were detected by camera-traps in the sampling area, size, shape, smell, composition (i.e. hair and parts of bones) and location of faeces were used to discriminate wolf and pet/hunting dog faeces [7–28]. The faecal samples collected were deep-frozen at −80 °C for at least seven days for biosecurity reasons (inactivation of Taeniidae eggs, including *E. granulosus s.l.* and *E. multilocularis*) before performing the parasitological analysis [13].

An aliquot of 2 g of each faecal sample was placed in 15 mL tubes and used for Taeniidae egg isolation. In order to obtain a representative aliquot of each faecal sample collected, portions from the central part and the extremities were isolated. Taeniid eggs were isolated using flotation with zinc chloride (ZnCl_2_) (specific gravity 1.350 solution) and the sieving method [38], and the morphological identification was carried out under a light microscope. During microscopic examination, co-infection by other parasites was also assessed.

### Molecular analysis

Genomic DNA extraction and PCR amplification were performed on faecal samples that were found to be positive for taeniid eggs at the parasitological analysis. For DNA extraction, each faecal sample was suspended in PBS 1X, filtered and centrifuged at 13,000 rpm for 10 min. The pellet obtained was then digested using 15 mg/mL proteinase K solution at 58 °C overnight and submitted to DNA extraction using a Fecal DNA kit (Bioline, United Kingdom), according to the manufacturer’s protocol. Partial sequences from the *cox1* and *nad1* gene marker were amplified following the PCR protocols proposed by Bowles et al. [5], Hüttner et al. [31], and Massolo et al. [37], including in each run, negative (ddH_2_O) and positive controls (*Echinococcus granulosus* DNA previously confirmed, Hammad et al. [27]). The amplicons were then purified and sequenced using Sanger automated sequencing by Bio-Fab Research (Rome, Italy). The resulting chromatograms were analysed and edited using Chromas v. 2.33 (Technelysium Pty Ltd, Australia). Readable sequences of partial *cox1* and *nad1* were aligned using MEGA7 [34] and compared to GenBank retrieved homologous sequences (https://www.ncbi.nlm.nih.gov/genbank/).

## Results

Overall, 38 wolf faecal samples were collected, of which 10.5% (4/38) were genetically characterized as belonging to three Italian wolf individuals. Microscopic examination evidenced taeniid eggs in 34.2% (13/38) of wolf faeces. Taeniid eggs were found in all samples belonging to the three genetically identified wolves. Taeniid DNA was successfully amplified with at least one target (*cox1* or *nad1*) in 10 out of 13 faecal samples containing taeniid eggs. Sequence analyses identified *Echinococcus granulosus s.s.* in 26.3% (10/38) and *Taenia hydatigena* in 10.5% (4/38) of analysed samples. All four positive samples for *Taenia hydatigena* showed co-infection with *Echinococcus granulosus s.s.*, confirmed by the same identity score for the *nad1* sequences from the two parasites. Moreover, under microscopic examination, *Capillaria* spp., Ancylostomatidae and *Toxocara canis* eggs, *Crenosoma vulpis* larvae, and coccidian oocysts were also detected (Table 1). Multiple parasite infections were found in 14/38 (36.84%) samples, while co-infestation of taeniid eggs with other parasites was detected in 8/38 (21.05%) positive samples (Table 2).

**Table 1:** Number of positive faecal samples for each identified parasite taxon.

Parasite	No. positive (%)
*Capillaria* spp.	21/38 (55.26%)
Ancylostomatidae eggs	7/38 (18.42%)
*Crenosoma vulpis* larvae	5/38 (13.15%)
Taeniidae eggs	13/38 (34.21%)
*Toxocara canis*	2/38 (5.26%)
Coccidian oocysts	1/38 (2.63%)

**Table 2:** Multiple parasite infections found in the wolf faecal samples analysed.

Samples	Taeniidae eggs	*Capillaria* spp.	Ancylostomatidae eggs	*Crenosoma vulpis* larvae	*Toxocara canis*	Coccidian oocyst
1		X	X			
2	X	X	X			
3		X	X			
4	X	X				
5	X	X				
6	X	X				
7	X		X		X	X
8		X	X			
9		X		X		
10	X	X				
11	X	X				
12			X		X	
13		X		X		
14	X	X	X			

## Discussion

In this study, the taeniid species occurring in a wolf pack living in a highly anthropic, hilly, agro-ecosystem were investigated for the first time. Taeniidae eggs were detected in 34.2% of analysed samples. This result is consistent with the frequency detection of Taeniidae eggs in faecal samples of wolves living in the northern Apennines and southern Alps in Liguria reported by Gori et al. [22] (33%, *n* = 179), in Foreste Casentinesi National Park by Poglayen et al. [49] (*n* = 42.1%, *n* = 130), and higher than those reported by Massolo et al. [37] (11.66%, *n* = 120) in the south-western Italian Alps.

Molecular amplification and identification by sequencing of taeniid species was obtained from 10 out of 13 faecal samples microscopically positive for Taeniidae eggs, while in the remaining three samples, no amplifiable products were obtained. This latter result is likely due to the sampling method as also reported in other studies [26, 52]. Importantly, the collection of faeces from the environment, as performed in this survey, represents a significant limiting factor in the extraction of high-quality target DNA in sufficient quantities. A long interval between faeces deposition on the soil and their collection can degrade the nucleic acid and increase the level of contamination by environmental organisms [52]. Such factors strongly reduce the possibility of DNA extraction and successful PCR amplification.

Sequence analyses of DNA extracted from faecal samples positive to taeniid eggs on microscopic examination allowed us to identify *E. granulosus s.s*. and *T. hydatigena*. However, the presence of other taeniid tapeworms in the analysed faecal samples cannot be ruled out, because the molecular protocol followed in this study preferentially amplified the predominant taeniid species. Cloning of the PCR product or high-resolution melting analysis is required to discriminate different species in the same host [9].

In this study, *E. granulosus s.s*. and *T. hydatigena* were detected in 26.3% (10/38) and 10.5% (4/38) of analysed faecal samples, respectively. In the wolf populations living in a natural landscape, *E. granulosus s.l*. has been detected in wolf faecal samples with a frequency of 5.6% (*n* = 179) in the Northern Apennines-Southern Alps in Liguria [22] and in the Apennine chain, with a prevalence of 17% (n=89 wolves) [23] and 15% (*n* = 119 wolves), respectively [24]. Conversely, *E. granulosus s.s*. was found in wolf faecal samples living in the Foreste Casentinesi National Park (2.3%, *n* = 120), with a prevalence of 5.5% (*n* = 54 wolves) [49] and *E. granulosus* (ovine genotype G1) was detected in Majella National Park in Abruzzo region with a prevalence of 5% (*n* = 20 wolves) [11]. *Taenia hydatigena* in wolves has been reported in mountainous, wild areas in the Northern Apennines-Southern Alps in Liguria and in the Apennine chain, with a frequency of 19.6% (*n* = 179) and a prevalence of 47% (*n* = 89) respectively, while in the Foreste Casentinesi National Park with a frequency of 23.8% (*n* = 120) [22, 23, 49].

The detection of *E. granulosus s.s.* as the main taeniid species and the detection of *T. hydatigena* in only a few samples in this wolf pack, in this specific, highly anthropic area, are particularly interesting compared to the high taeniid biodiversity detected in wolf populations living in wild mountain areas of the Apennines [22, 23, 49], as well as in other European countries [4, 25, 43] (Table 3). Also in wild, mountainous areas of the Alps, higher taeniid biodiversity was recorded compared to that detected in this study [37]. In this case, it is important to point out that this difference could be due also to the different taeniid DNA extraction protocols used, from faecal samples in this study and from taeniid eggs by Massolo et al. [37]. In wild habitats, the wolf was identified as the definitive host of several Taeniidae species, among which *T. hydatigena* was the most frequently detected species followed by *T. multiceps*, *T. ovis*, *T. krabbei*, *T. pisiformis*, *T. crassiceps*, *T. polyacantha*, *E. ortleppi*, *E. multilocularis* and *E. granulosus*, suggesting a more diversified multi-host parasite system [22, 23, 37, 49]. All these Taeniidae species are recorded throughout Europe in several intermediate hosts, such as large ungulates, lagomorphs and small mammals [36, 45, 46, 55].

**Table 3:** Taeniid biodiversity detected in different wolf populations living in the Apennines and Southern Alps in Italy and in other European countries. For each Taeniidae, the frequency or prevalence detected are reported if provided in the respective literature sources.

Taeniid species	Area	Reference
*Taenia hydatigena* (47%)*	Apennine chain	Guberti et al. [23]
*Taenia multiceps* (9%)*		
*Taenia pisiformis* (7%)*		
*Taenia ovis* (3%)*		
*Echinococcus granulosus s.l.* (17%)*		
*Taenia hydatigena* (40.7%)**	Northern Apennines–Southern Alps (Liguria)	Gori et al. [22]
*Taenia ovis* (2.2%)**		
*Taenia krabbei* (4.5%)**		
*Taenia crassiceps* (0.6%)**		
*Hydatigera taeniaeformis* (0.6%)**		
*Echinococcus granulosus s.l*. (5.6%)**		
*Taenia ovis*	Modena Apennines	Fiocchi et al. [18]
*Taenia hydatigena*		
*Taenia hydatigena* (40.7%)*	Foreste Casentinesi National Park, Northern Italian Apennines	Poglayen et al. [49]
*Taenia krabbei* (22.2%)*		
*Taenia polyachantha* (1.8%)*		
*Echinococcus granulosus s.s*. (5.5%)*		
*Taenia hydatigena*	Ligurian Alps Regional Park, Italian Maritime Alps	Massolo et al. [37]
*Taenia krabbei*		
*Taenia ovis*		
*Taenia multiceps*		
*Echinococcus ortleppi*		
*Echinococcus multilocularis*		
*Taenia hydatigena* (12%)*	Estonia	Moks et al. [43]
*Taenia multiceps* (27%)*		
*Taenia ovis* (15%)*		
*Taenia pisiformis* (8%)*		
*Echinococcus granulosus s.l*. (4%)*		
*Taenia hydatigena* (13.3%)**	Germany	Bindke et al. [4]
*Taenia krabbei* (13.3%)**		
*Taenia hydatigena* (11.8%)**	Portugal	Guerra et al. [25]
*Taenia serialis* (5.9%)**		
*Taenia pisiformis* (2.9%)**		
*Taenia polyachantha* (1.5%)**		
*Echinococcus intermedius* (G7) (1.5%)**		

* Percentage refers to a prevalence.

** Percentage refers to a frequency.

*Echinococcus granulosus s.l*. is widespread in the Mediterranean basin, especially in Spain and Italy (southern Italy and Sardinia), with over 1000 human cystic echinococcosis (CE) cases per year in both countries [10, 50]. The life cycle of *E. granulosus s.s* is mostly domestic or semi-domestic, involving dogs as the most important definitive host, and wolves and domestic ungulates as intermediate hosts [51]. Sheep and more rarely goats are the most important domestic intermediate hosts for *E. granulosus s.s*, which may also infect other herbivorous hosts (e.g. cattle, camels, donkeys and macropods) [51]. *Taenia hydatigena* is a very generalist taeniid species, which parasitises as the larval stage (*Cysticercus tenuicollis*) a wide range of intermediate hosts, both domestic animals (i.e. sheep) and wild ungulates (i.e. mouflon, red deer, roe deer, fallow deer and wild boar) [19, 36, 45, 48, 53, 54].

The highly anthropic, sub-urban, hilly area in which this study was performed is characterised by the presence of extensive sheep farming, the availability of wild prey, and close contact between domestic animals, humans and wildlife. These features make this area completely different from the Alps and Apennine mountain habitats. In anthropic rural areas, livestock (i.e. sheep and goats), which are easily accessible and highly vulnerable, and wild ungulates, which recently increased their diffusion in urbanised areas are an important source of food for wolves [40, 41]. In the study area, wolf attacks on sheep and goats were regularly recorded during the investigation period (unpublished data). No data are available concerning the frequency of livestock and wild prey in the wolf diet in anthropic areas, and there is a lack of information on the frequency and prevalence of echinococcosis and cysticercosis in livestock and wildlife in anthropic agro-ecosystems.

Therefore, the high detection of *E. granulosus s.s*. found in this investigation should be ascribed to the highly anthropised environment with a high livestock density. No wild cycle of *E. granulosus s.l.* has been described in Italy, even though,the wild boar was recently found to be a wild intermediate host of *E. granulosus s.l*. and *Taenia hydatigena* [12, 35, 48, 58]. Moreover, differences occurring in taeniid biodiversity in wolves living in anthropic areas could be the result of a combined effect of a low occurrence of taeniids in wild prey and a lower frequency of wild prey in the diet of wolves in anthropic areas than in those living in natural landscapes.

All four positive samples for *Taenia hydatigena* in this study showed co-infection with *E. granulosus s.s*. Taeniid co-infection in wild wolf populations was previously reported with *T. hydatigena* and *E. granulosus s.l*. by Gori et al. [22] and with *E. multilocularis* and *T. hydatigena*, *T. krabbei*, *T. ovis*, or *E. ortleppi* by Massolo et al. [37]. Moreover, co-infection with *T. hydatigena* and *E. granulosus s.l*. was also recorded in wild boar hunted in two National parks in Central Italy [48].

In this study, microscopic examination also demonstrated the occurrence of *Capillaria* spp., Ancylostomatidae eggs, *Crenosoma vulpis* larvae*, Toxocara canis* and coccidian oocysts, as previously detected in wolves both from Italy and other countries in Europe [2, 17, 18, 23, 30, 44].

In conclusion, the results obtained in this investigation support the prediction that: (i) *E. granulosus s.s*. is the most frequently recorded taeniid species in wolves living in highly anthropised, hilly agro-ecosystems as a consequence of a deeper link occurring between wolves and livestock, (ii) taeniid biodiversity is lower than in wolves living in wild habitats, even though this result needs to be reinforced by further investigations using DNA extraction directly from taeniid eggs, rather than from faecal samples. Moreover, the results obtained indicate that co-infection with several taeniid species occurred in wolves. Further studies are needed in order to assess the taeniid species frequency occurring in wolves from other anthropised areas. In addition, investigations on wolf diet composition in anthropic areas compared to that of wild individuals would be useful.

## Funding and source

This study was funded by Fondi di Ateneo of the University of Pisa, Italy.

## Conflict of interest

The authors declare that they have no conflict of interest.

## Author contributions

Conceived the study: AF, FM, FC; Designed the experiment: AF, FM, FC; Performed the field activities and sampling: AF, FC, SB, CBB; Performed the laboratory work: FM, FF, SG; Analysed and interpreted the data: AF, FM, FC, FF, SG, SB, CBB; Wrote the original draft of the manuscript: AF, FM, FC, FF, CBB; Reviewed and edited the final version of the manuscript: AF, FM, FC, SG, FF, SB, CBB; Supervision: AF.
